# Quantitative analysis of computed tomography images and early detection of cerebral edema for pediatric traumatic brain injury patients: retrospective study

**DOI:** 10.1186/s12916-014-0186-2

**Published:** 2014-10-22

**Authors:** Hakseung Kim, Gwang-dong Kim, Byung C Yoon, Keewon Kim, Byung-Jo Kim, Young Hun Choi, Marek Czosnyka, Byung-Mo Oh, Dong-Joo Kim

**Affiliations:** Department of Brain and Cognitive Engineering, Korea University, Anam-dong, Seongbuk-gu, Seoul, 136-713 South Korea; Department of Rehabilitation Medicine, Seoul National University College of Medicine, Seoul, South Korea; Department of Neurosurgery, Stanford University School of Medicine, Stanford, California USA; Department of Neurology, Korea University College of Medicine, Seoul, South Korea; Department of Radiology, Seoul National University Children’s Hospital, Seoul, South Korea; Academic Neurosurgical Unit, University of Cambridge Clinical School, Cambridge, UK

**Keywords:** Cerebral edema, Computed tomography, Densitometry, Traumatic brain injury, Pediatrics

## Abstract

**Background:**

The purpose of this study was to identify whether the distribution of Hounsfield Unit (HU) values across the intracranial area in computed tomography (CT) images can be used as an effective diagnostic tool for determining the severity of cerebral edema in pediatric traumatic brain injury (TBI) patients.

**Methods:**

CT images, medical records and radiology reports on 70 pediatric patients were collected. Based on radiology reports and the Marshall classification, the patients were grouped as mild edema patients (n = 37) or severe edema patients (n = 33). Automated quantitative analysis using unenhanced CT images was applied to eliminate artifacts and identify the difference in HU value distribution across the intracranial area between these groups.

**Results:**

The proportion of pixels with HU =17 to 24 was highly correlated with the existence of severe cerebral edema (*P* <0.01). This proportion was also able to differentiate patients who developed delayed cerebral edema from mild TBI patients. A significant difference between deceased patients and surviving patients in terms of the HU distribution came from the proportion of pixels with HU = 19 to HU = 23 (*P* <0.01).

**Conclusions:**

The proportion of pixels with an HU value of 17 to 24 in the entire cerebral area of a non-enhanced CT image can be an effective basis for evaluating the severity of cerebral edema. Based on this result, we propose a novel approach for the early detection of severe cerebral edema.

## Background

Increased intracranial pressure (ICP) is often observed in traumatic brain injury (TBI). Elevated ICP results in decreased cerebral perfusion pressure (CPP), which can cause cerebral ischemia. One of the most frequent effects of cerebral ischemia is cerebral edema, which is a major cause of brain swelling [1-4]. Swollen parenchymal tissue may compress capillaries, aggravating cerebral ischemia by further decreasing the cerebral blood flow (CBF) [5-7]. Computed tomography (CT) and magnetic resonance imaging (MRI) are widely used to assess the extent of brain injury in TBI. MRI offers greater resolution and sensitivity for the detection of cerebral edema than CT [8]. By taking the apparent diffusion coefficient (ADC) in diffusion-weighted MRI (DWI), it is possible to achieve even higher sensitivity in detecting cerebral edema [9-12]. However, the higher sensitivity of MRI does not lead to significant changes in the management of TBI [13-15]. MRI also takes significantly more time than CT and requires patients to lie still during the examination, which can be particularly challenging in pediatric patients. For this reason, sedation or even generalized anesthesia for MRI is often used in pediatric patients [16]. Two major complications of generalized anesthesia in pediatric patients are cardiac and respiratory events [17]. Combined with the damage caused by the primary injury, these complications may further aggravate the secondary insults.

Compared to MRI, CT offers greater accessibility with faster image acquisition time, which can be particularly advantageous in acute settings with unstable patients. Despite these advantages, the utility of CT has been questioned due to the interpretation of its images, particularly in regard to the detection of cerebral edema, as it relies heavily on the radiologist’s subjective impressions of subtle differences in attenuation [18]. To standardize CT interpretation, several CT classifications for brain injury have been devised, mainly to assess the severity of stroke. Some of these systems may be feasible for assessing the severity of TBI because ischemic stroke and TBI share similar mechanisms [19]. More than ten TBI classification systems using CT have been proposed to date [20]. However, no classifications have yet been as heavily evaluated as the Marshall classification [21]. The Marshall classification uses CT findings regarding structural abnormalities, such as the compression of basal cisterns, a shift of the midline and the volume of traumatic lesion. This classification gives simple ordinal scores ranging from 1 to 6 and is known for showing good correlation with the outcome [22]. The Marshall classification provides a significant amount of prognostic information in TBI. Nevertheless, the Marshall classification may need further refinement [23]. The Marshall classification has been criticized for its low sensitivity to neurophysiological changes and significant intra- and inter-rater variability; most of all, it was not designed for assessing the severity of cerebral edema. Another major limit of existing CT classifications is that they may not reflect characteristic aspects of the pediatric brain.

More than 60% of pediatric TBI patients exhibit increased ICP [24]. Immature brains are more vulnerable to increased ICP than adults as a result of their lower intracranial compliance [25]. As a consequence, the mortality rate of pediatric patients is threefold higher than in adults [6]. While an increase in CBF also causes brain swelling, recent studies suggest hyperemia is not a major cause of brain swelling in pediatric patients [26,27]. In fact, decreased CBF is common in pediatric TBI patients [28]. Cerebral edema is thus considered the major cause of increased ICP in pediatric TBI cases. The presence of cerebral edema is assessed by parenchymal hypoattenuation in a CT image. In this study, we hypothesized that the proportion of pixels with specific attenuation levels can be used to detect the presence of cerebral edema. A quantitative standard can compensate for the weakness of the Marshall classification, which relies on the subjective interpretation of images. Furthermore, certain TBI patients may later develop delayed cerebral edema. Early quantitative signs of delayed cerebral edema may exist in the initial CT images. The early detection of cerebral edema from initial CT images may be possible using the quantitative standard. We performed this study to investigate whether the proportion of pixels with specific attenuation levels in brain CT images can be used to detect the presence of cerebral edema and to develop a quantitative standard for rapid assessment of the severity of cerebral edema in pediatric patients.

## Methods

The difference in density distribution between CT images from patients with severe cerebral edema and patients with mild cerebral edema was investigated. For an accurate comparison, the CT images had to be standardized. A tool for automated artifact elimination and densitometric measurement was devised. After the images were processed and the density distribution had been obtained, statistical analyses were employed to find the pixels that caused the difference between density distributions of the two groups. The proportion of these pixels in a set of CT images was defined as the cerebral edema score (CES). The CES from deceased patients was defined as mortality-related CES (mCES). Additionally, it is well known that the brain water content varies with age [29,30]. The difference caused by age is clearly recognizable after the first two years of life [31,32]. The age-induced difference in the density distribution was also investigated to determine whether the difference between the severe edema group and the mild edema group was affected by the age factor.

### Subjects

The medical records and CT images of 70 pediatric TBI patients were collected for this study (Table 1). The subjects had been admitted to the Seoul National University Hospital (SNUH), Seoul, Korea from 2003 to 2013. The use of the patient material was approved by SNUH (IRB H-1402-020-553). The primary classification of subjects was performed via radiology reports. The existence of cerebral edema and the extent of the resulting damage were confirmed by reports and CT images. Pediatric TBI patients are highly susceptible to the development of cerebral edema. Furthermore, the subtle hypoattenuation caused by ischemic change is often difficult to detect [29]. To minimize methodological complications, detailed severity measures were not taken. Instead, this study dichotomized subjects into two groups by the Marshall classification. Marmarou *et al*. reported that the brain water content of TBI patients with Marshall scores I and II is close to normal [30]. Based on their findings, we applied the Marshall Classification system to differentiate the edematous subjects into severely edematous (Marshall score III or higher) and mildly edematous (Marshall score I or II). Some subjects with no clear evidence of cerebral edema on initial CT developed severe cerebral edema 12 hours later; they were designated as delayed cerebral edema subjects and considered severely edematous.

**Table 1:** **Baseline characteristics of study subjects**

	**Total**	**Mildly edematous (n = 37)**	**Severely edematous (n = 33)**
**Death (%)**	**6 (8.6)**	**0 (0)**	**6 (18.2)**
Cause of trauma			
MVA	12 (17.1)	1 (2.7)	11 (33.3)
Fall	15 (21.4)	5 (13.5)	10 (30.3)
Other	43 (61.4)	31 (83.8)	11 (36.4)
Marshall Score			
Grade I	34 (48.6)	33 (89.2)	1 (3.0)
Grade II	6 (8.6)	4 (10.8)	2 (6.1)
Grade III	9 (12.9)	0 (0)	9 (27.3)
Grade IV	1 (1.4)	0 (0)	1 (3.0)
Grade V	0 (0)	0 (0)	0 (0)
Grade VI	20 (28.6)	0 (0)	20 (60.6)

The 2nd column contains general demographics, while the 3rd and 4th columns contain detailed information about two subgroups (mildly edematous patients and severely edematous patients). The Marshall score was obtained from the first CT scan image; the three severely edematous subjects who showed a Marshall score less than 2 later developed severe cerebral edema. MVA, motor vehicle accident.

### Image processing

The scans were performed on a Brilliance 64 CT scanner (Philips Healthcare, Best, Netherlands). As in many institutions, the brain images were acquired in non-helical mode [31], thereby effectively avoiding unnecessarily high exposure to radiation and image artifacts. Contiguous, unenhanced 5 mm-slice axial CT images were used for analysis. The images used were obtained within 24 hours after hospital arrival. The image matrix sizes were 512 by 512; X-ray tube voltage, 120 kVp; X-ray tube current, 120 to 250 mA; exposure, 120 to 250 mAs. The median CT dose index volume (CTDIvol) was 21.3 mGy (range = 16.0 to 35.4 mGy), and the median dose length product (DLP) was 372.4 mGy · cm (range = 216.7 to 805.3 mGy · cm). The reconstruction kernel was set as ‘UB’ (for standard brain imaging). Continuous axial sections from the vertex to the caudal end of the cerebrum were collected for each patient. Artifact elimination and densitometric measurements were performed by in-house software built using MATLAB (MathWorks Inc., Natick, MA, USA).

**Figure 1:**
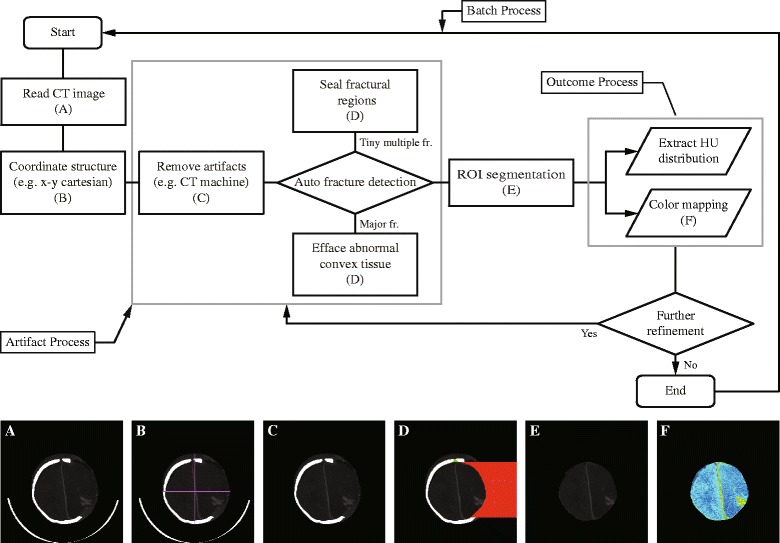
**An algorithm for automated artifact elimination.** The program successfully removes EVD, ICP monitors, air bubbles, the cranium, or any other artifacts that might corrupt the densitometry analysis. The algorithm works in a sequential order **(A–F)**, and the resulting image can be used for histogram analysis: **(A)** Reading of a CT image. **(B)** Coordination of cranial structure. **(C)** Removal of obvious artifacts. **(D)** Sealing fractural regions. **(E)** Elimination of convex tissue. **(F)** ROI segmentation in accordance with the pre-designated boundary condition. CT, computed tomography; EVD, external ventricular drain; ICP, intracranial pressure; ROI, region of interest.

A tool was designed for the rapid densitometry analysis of CT images. The algorithm regards every pixel outside the cranium as an artifact and can thus quickly dispose of pixels unnecessary for the analysis (Figure 1). To analyze only the intracranial components in CT images, the pixels representing the cranium, soft tissues outside the cranium, and other artifacts, such as foreign bodies or artifacts generated by the CT machine itself, should be eliminated. The cranium pixels and foreign body pixels can be easily eliminated by using their Hounsfield Unit (HU) values. Eliminating the soft-tissue pixels outside the cranium can be more complicated, as the HU value range of soft-tissue pixels is similar to the range of intracranial component pixels. Although they can be differentiated by their relative positions (outside versus inside the cranium), employing such a method for CT images of a fractured cranium can cause an erroneous result. As a preemptive measure to avert such an error, this algorithm seals such fractures by temporarily generating pseudo-cranial pixels. Once a CT image is read (Figure 1A), the algorithm assigns four quadrants to the intracranial area (Figure 1B). These quadrants identify fractures generated by the impact of the primary injury, or craniotomy. Before proceeding to the next step, pixels representing the CT machine itself are eliminated (Figure 1C). Multiple fractures along the cranium can be developed due to the impact of the primary injury. Such fractures are defined as a discontinuation of the cranial pixels and can be sealed off by creating pseudo-cranial pixels (Figure 1D). Sizable fractures, common in CT images after craniotomy, cannot be handled by this method. The algorithm seals them off using the pseudo-axisymmetric property of the human cranium (Figure 1D). It then removes any pixels with a density outside the pre-designated boundary condition (Figure 1E). More details of the process are given in the Appendix. The algorithm allows further post-processing such as densitometric measurements and color mapping. The results are modified CT images composed of pixels depicting only parenchyma, cerebrospinal fluid (CSF) and blood (Figure 1F). The use of this algorithm allows fully automated rapid densitometry analysis for vast numbers of CT images.

### Densitometric analysis

The HU values of major intracranial components in a CT image generally do not exceed 80 nor fall below 0 [32,33]. Based on this fact, attenuation threshold limits of 0 to 79 HU were used. Any pixels with an HU value outside this boundary condition were excluded from the analysis. Each pixel in a CT image has a certain HU level, λ, which is between 0 and 79 in this study. The proportion of specific pixels with λ HU in a CT image can be obtained by simple arithmetic. Let λ_*p*_ be the proportion of pixels with λ HU in a CT image; then,

$$ 0\le \uplambda \le 79,\kern1em 0\le {\uplambda}_p\le 100,\;\mathrm{and}\ {\displaystyle \sum_{\Delta =0}^{79}{\uplambda}_p}=100. $$

The proportion (%) of λ in a CT image is 0 to 100, with the sum of every λ_*p*_ as 100, as per the definition of λ_*p*_. In reality, a CT examination on a TBI patient produces a series of images. If a CT examination generates *k* number of CT images, there will be *k* number of λ_*p*_ for each image. Let ${\uplambda}_p^k$ be the proportion of pixels which have HU = λ in every pixel in the *k*^th^ CT image in a given set. Then, the proportion of pixels with HU = λ in an entire pixel of the set can be expressed as $\frac{1}{n}{\displaystyle \sum_{k=1}^n{\uplambda}_p^k}$ = the average λ_*p*_ in a number *n* of CT images, where $\frac{1}{n}{\displaystyle \sum_{k=1}^n{\displaystyle \sum_{\Delta =0}^{79}{\uplambda}_p^k}}$ = 100, and *n* is the total number of images generated by the CT examination. The density distribution of a subject can be obtained by plotting $\frac{1}{n}{\displaystyle \sum_{k=1}^n{\uplambda}_p^k}$, for every λ. If there is a significant difference in density distribution between two sets of CT images, the difference can be expressed as the proportion of specific pixels. The proportion of pixels with HU = α to β in a set of CT images can be easily denoted as $\frac{1}{n}{\displaystyle \sum_{k=1}^n{\displaystyle \sum_{\Delta =\upalpha}^{\upbeta}{\uplambda}_p^k}}$. For convenience, $\frac{1}{n}{\displaystyle \sum_{k=1}^n{\displaystyle \sum_{\Delta =\upalpha}^{\upbeta}{\uplambda}_p^k}}$ is hereafter denoted as ${\mathrm{HU}}_{\upalpha}^{\upbeta}$.

### Statistical analysis

Statistical analyses were conducted using commercial software (SPSS version 21; IBM) to obtain the baseline characteristics of the subjects and compare averaged density distributions between the subject groups to find the difference in terms of the proportion of specific pixels. Shapiro-Wilk tests showed that normality cannot be assumed. Non-parametric statistical analyses were used for this reason. The median value, range and percentages were selected as descriptive statistics. The Kruskal-Wallis and Mann–Whitney U tests were conducted to compare the average density distributions of subject groups. With pixels having a specific HU value that caused a significant difference between the density distributions identified, the average proportions of these pixels were obtained. A receiver operator characteristics (ROC) curve determined the cutoff value of the proportions. Pearson’s Chi-square or Fisher’s exact test were applied for categorical variables. A statistically significant result was defined as a *P*-value of less than 0.05.

## Results

A total of 70 TBI patients’ data were used, and 42 (60%) subjects were males. The median age of the subjects was 37 months (range = 2 to 224 months); 33 (47.1%) subjects were classified as severely edematous. Among those subjects, eight (24.20%) were not initially diagnosed as edematous but later developed delayed cerebral edema (mean onset time of edema detection after admission = 36.33 hours). Six patients died during hospitalization. Every deceased patient had CT signs of cerebral edema. Only one patient had had surgery before being subjected to CT examination. The remaining details are listed in Table 1.

### Comparison of mild and severe edema subjects

**Figure 2:**
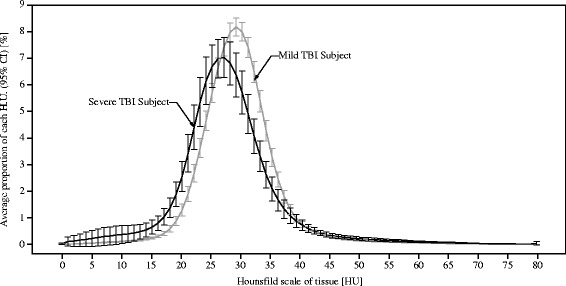
**A graphical comparison of mild and severe TBI subjects in terms of HU value distribution.** Black = severe TBI subjects, gray = mild TBI subjects. HU, Hounsfield Unit; TBI, traumatic brain injury.

**Figure 3:**
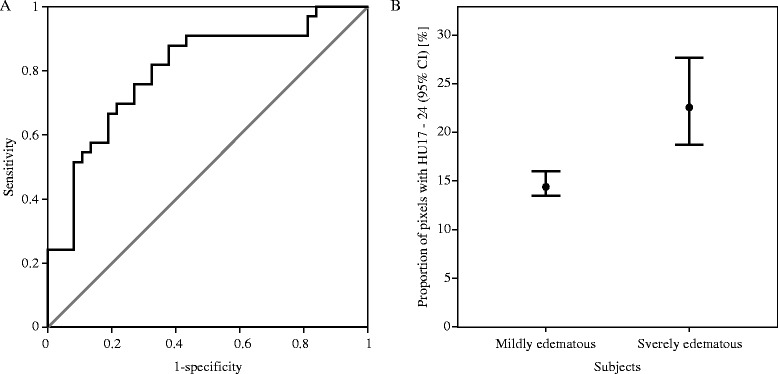
**The determinative power of the proportion of** ${\mathbf{HU}}_{\mathbf{17}}^{\mathbf{24}}$ **in differentiating severe and mild edema subjects. (A)** A non-parametric ROC curve. Area under the curve = 0.85, with asymptotic *P* = 0.005. The cutoff point was set to 22.58% – giving a sensitivity of 0.82 and a specificity of 0.68. Gray = reference line, black = empirical ROC curve. **(B)** An error bar chart for comparing mildly edematous subjects and severely edematous subjects in terms of the proportion of pixels with 17 to 24 HU. HU, Hounsfield Unit; ROC, receiver operator characteristics.

The average density distributions of mild (n = 37) and severe edema subjects (n = 33) were compared. A density histogram depicts the difference between these two groups (Figure 2). The overall CT image density of severe edema subjects was lower in mild edema subjects. A major difference between the two groups was the proportion of pixels with 17 to 24 HU (${\mathrm{HU}}_{17}^{24}$, *P* <0.0001). The median values of ${\mathrm{HU}}_{17}^{24}$ of the mild edema group and the severe edema group were 14.31 and 22.48, respectively. Subjects with severe edema showed a higher proportion of ${\mathrm{HU}}_{17}^{24}$ in their CT image than mild edema subjects. An ROC curve and error bar chart were plotted to verify the determinative power of ${\mathrm{HU}}_{17}^{24}$ (Figure 3). The area under the curve was 0.85, with an asymptotic *P* = 0.005. The cutoff point was determined to have the highest predictive power. ${\mathrm{HU}}_{17}^{24}$ greater than or equal to 16.03% (sensitivity = 0.82, specificity = 0.68) was highly correlated with severe cerebral edema. To further verify the credibility of ${\mathrm{HU}}_{17}^{24}$ as an indicator for severe edema, additional analyses were applied to confirm whether the age related changes in density distribution affected ${\mathrm{HU}}_{17}^{24}$. There were significant differences in density distribution between patients at or younger than 24 months of age (n = 22, median age = 8.50 months) and patients who were older than 24 months (n = 48, median age = 53.50 months), particularly caused by the proportion of pixels with 7 to 11 HU (${\mathrm{HU}}_7^{11}$, *P* <0.05), 23 to 28 HU (${\mathrm{HU}}_{23}^{28}$, *P* <0.05) and 32 to 47 HU (${\mathrm{HU}}_{32}^{47}$, *P* <0.05). Within the severely edematous patient group and the mildly edematous patient group, comparisons regarding age difference resulted in no significant difference in ${\mathrm{HU}}_{17}^{24}$ (*P* >0.5). Tests within each age group showed that the difference in density distribution between severe edema and mild edema was caused by the proportion of pixels having HU 21 to 23 (${\mathrm{HU}}_{21}^{23}$, *P* <0.05) in patients at or younger than 24 months of age and HU 13 to 24 (${\mathrm{HU}}_{13}^{24}$, *P* <0.05) in patients older than 24 months of age.

### Density distribution of subjects with delayed cerebral edema

**Figure 4:**
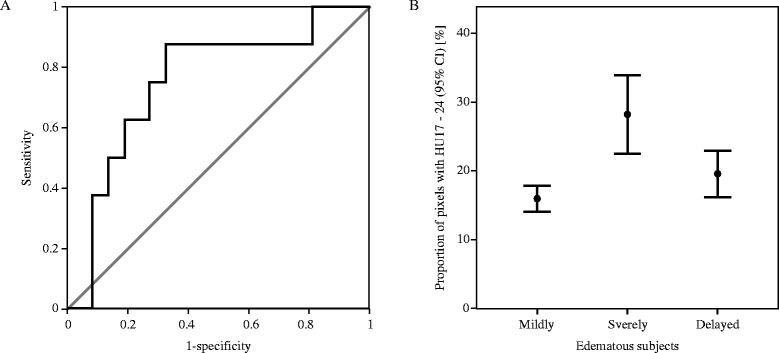
**The determinative power of** ${\mathbf{HU}}_{\mathbf{17}}^{\mathbf{24}}$ **in differentiating delayed edema subjects and mild edema subjects. (A)** A non-parametric ROC curve. Area under the curve = 0.75, with asymptotic *P* <0.03. The cutoff point was set to 16.29% – giving a sensitivity of 0.88 and a specificity of 0.68. Gray = reference line, black = empirical ROC curve. **(B)** An error bar chart depicting the proportion of pixels with 17 to 24 HU in CT images from three subject groups. Mildly = mild edema subjects. Severely = subjects with severe cerebral edema confirmed by initial CT examination. Delayed = subjects who developed delayed cerebral edema. CT, computed tomography; HU, Hounsfield Unit; ROC, receiver operator characteristics.

Among 33 severe edema subjects, 25 exhibited early CT signs of cerebral edema, while the other eight subjects did not. These subjects developed delayed cerebral edema, which was confirmed by later CT examinations due to their neurological changes. Density histograms of the initial CT images from these patients were very similar to the severely edematous group. The Kruskal-Wallis test proved a significant difference between mildly TBI, the severe edema group, and subjects with delayed cerebral edema. To further test the predictive power of ${\mathrm{HU}}_{17}^{24}$, a Mann–Whitney U test was applied to subjects with delayed cerebral edema and the mild edema group. Consequently, ${\mathrm{HU}}_{17}^{24}$ was identified as the cause of the difference between these two groups (*P* <0.03). Furthermore, the average proportions of ${\mathrm{HU}}_{17}^{24}$ in subjects with delayed cerebral edema were similar to the proportions in patients who had severe cerebral edema (Figure 4). ${\mathrm{HU}}_{17}^{24}$ greater than or equal to 16.29% (area under the curve = 0.75, sensitivity = 0.88, specificity = 0.68) was highly correlated with the development of delayed cerebral edema (Figure 4). The negative and positive predictive values of the threshold were 0.96 and 0.37, reflecting a low prevalence of delayed edema among the study subjects.

### Comparison of surviving and deceased subjects

**Figure 5:**
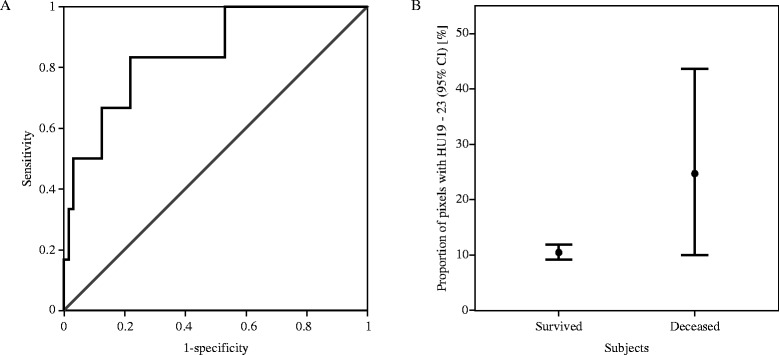
**The determinative power of the** ${\mathbf{HU}}_{\mathbf{19}}^{\mathbf{23}}$ **in differentiating deceased and surviving subjects. (A)** A non-parametric ROC curve. Area under the curve = 0.85, with asymptotic *P* = 0.005. The cutoff point was set to 15.27% – giving a sensitivity of 0.83 and a specificity of 0.78. Gray = reference line, black = empirical ROC curve. **(B)** An error bar chart for comparing deceased subjects and surviving subjects in terms of the proportion of pixels with 19 to 23 HU. HU, Hounsfield unit; ROC, receiver operator characteristics.

${\mathrm{HU}}_{19}^{23}$ proved to be highly significant in differentiating deceased subjects (n = 6) from surviving subjects. Asymptotic *P* values were less than 0.01 for ${\mathrm{HU}}_{19}^{23}$. ${\mathrm{HU}}_{19}^{23}$ greater than or equal to 15.27% (area under the curve = 0.85, sensitivity = 0.83, specificity = 0.78) was highly correlated with mortality (Figure 5). The peak of the average density histogram of deceased subjects was near HU24, whereas for surviving subjects it was near HU29.

## Discussion

Early studies suggested that immature brains were more prone to develop cerebral edema after TBI than adults [6,25,34]. Compared to adult brains, immature brains have a more fragile blood brain barrier and a higher brain water content. These characteristics may contribute to the rapid development of secondary insults from TBI [35]. This implies that the window of successful intervention for cerebral edema can be narrower in pediatric TBI patients than in adult TBI patients. The rapid detection and anticipation of cerebral edema is even more important in pediatric TBI patients.

### Technical implications

The difficulty of interpreting the result has always been a problem for CT imaging. Quantitative analysis for CT was devised to compensate for this difficulty. Quantitative CT (QCT) methods have beeen mainly used in other specialties, such as cardiology and pulmonology [36-43]. Recent neuroradiological studies, which used densitometry analysis, focused on finding tumors or regional pathological changes [44-48]. Only a handful of studies used densitometric methods for whole brain images of abnormal brains [49-52]. Although few in number, these studies show promising results. One of the main purposes of this study was to test the feasibility of automated densitometry analysis on CT images.

### Conventional problems regarding automated CT densitometry analysis

There are some prerequisite conditions for automating CT densitometry analysis. Diagnostically irrelevant objects, such as artifacts, the cranium, catheter/ICP monitor, air cells, image background, or the CT machine itself, should be eliminated prior to the densitometry analysis. These unwanted data can complicate quantitative analysis and result in misinterpretation. The use of different CT scanners, reconstruction kernels, or adjustments of the tube voltage or currents that can affect the HU value itself in CT imaging should also be addressed [53]. While the effect of scanner variation on quantitative CT analysis is controversial [53,54], the fluctuation in HU caused by acquisition parameters, such as the tube current and voltage, can be substantial [55]. To minimize HU fluctuation in CT, the tube voltage and current must be properly adjusted [54]. Although the HU values of soft tissues (HU in the range 0 to 100) do not show significant fluctuation, this CT-imaging characteristic can be an obstacle for automated CT image analysis [51]. The designation of ROI is another major concern in quantitative CT analysis. While the HU fluctuation from the use of different CT acquisition parameters can be prevented, the designation of ROI may not. The manual designation of ROI can be susceptible to inter-observer variability. If pre-analytic CT images are to be standardized, the designation of ROI should be automated.

### Automation of densitometric measurements through standardization

To address complications regarding automated densitometry analysis, the tool devised for this study has several distinctive functions. In CT imaging, the density of a material is defined as its relative ability to absorb X-ray photons. This ability is expressed as HU. The HU of certain important substances is well known. For instance, the HU of CSF is between 0 and approximately 15, normal cerebral tissue has an HU of less than 40, and the HU of hemorrhagic cells is below 80 [32,33]. For the purpose of this study, any objects other than these three are considered artifacts. For this reason, attenuation threshold limits of 0 to 79 were used in this study. With these limits, artifacts were automatically eliminated. The threshold limits of 0 to 79 enabled the standardization of pre-analytic CT images without compromising the data integrity. Standardization of the CT images allows automated densitometry analysis. The tool devised for this study reads a set of images, eliminates artifacts and leaves only pixels with HU = 0 to 79. The tool then calculates the average proportions of pixels with HU 0, 1, … 79 in the set of images. The result is the density distribution of the entire cerebral area of a subject. Inter-observer variability is minimized with the automation of densitometric measurements. The algorithm employed by the tool enables continuous, automated CT densitometry analysis for groups of images, which allows the density distribution of a group of subjects to be obtained within a few minutes. After the density distributions of groups A and B are calculated, statistical analysis of the distributions can reveal the source of the significant difference between groups A and B. This difference was expressed as ${\mathrm{HU}}_{\upalpha}^{\upbeta}$, the proportion of pixels with HU = α to β in a set of CT images. By comparing proportions, the influence of HU variability was minimized.

### Clinical significance

The exact mechanisms of cerebral edema are not well understood. Understanding cerebral edema from TBI is even more challenging given its heterogeneity. Researchers have focused on the molecular mechanisms of cerebral edema [56-58], which are often difficult to translate into a clinical setting [22]. Currently, the Brain Trauma Foundation recommends ICP-oriented therapy for pediatric TBI patients. Repeat CT scans are considered when ICP therapy does not seem to be effective [59]. Brain CT densitometry analysis can provide a rapid assessment of pathological changes in an abnormal brain.

### Quantitative severity assessment for cerebral edema

While there are notable quantitative brain CT analysis studies on cerebral edema or TBI [47,60], most prior studies on the quantitative assessment of cerebral edema used mean HU values in a pre-designated edematous region as the subject of analysis. However, such approach may not have the desired diagnostic value for several reasons. The designation of ROI is susceptible to inter-observer variability. Inaccurate segmentation of an edematous lesion would result in erroneous interpretation of the CT image. Moreover, the designation of ROI itself can be complicated in diffuse cerebral edema. The ‘mean HU value of the ROI’ approach, for these reasons, may not be appropriate for quantitative CT analysis of pediatric cerebral edema. Alternatively, other studies have used densitometry analysis, based on predefined values, on pixels of edematous tissue. Rosza *et al*. separated diffuse brain swelling patients into edema and hyperemia groups according to the number of pixels of specific HU [49]. In this study, pixels having an HU between 11 and 20 were considered edematous pixels. Mangel *et al*. made a similar assumption, where an edematous pixel was defined as having a HU of 10 to 28 [48]. While intuitive, results from these predefined definitions may not reflect the actual damage caused by cerebral edema. This inherent limitation becomes more problematic when applied to pediatric patients, as these patients have a higher brain water content than adults. To address these problems, standardization of CT images and whole brain analysis were employed in this study. Rather than analyzing pre-designated pixels of interest, we compared the density distributions of the whole cerebrum for severely edematous patients and mildly or moderately edematous patients. This method revealed the actual difference between CT images of severely edematous brains and mildly edematous brains, allowing a standardized global assessment of cerebral edema.

### Cerebral edema score

Subjects with severe cerebral edema showed a lower overall density than mild edema subjects. The average density histogram for the two groups clearly illustrates this difference (Figure 2). Compared to the mild edema group, subjects with severe cerebral edema exhibited an increased proportion of pixels having HU 17 to 24 (${\mathrm{HU}}_{17}^{24}$) in an entire set of CT images for the CT examination.

It is well known that CT attenuation and brain water content are inversely correlated [61]. The age related difference in image attenuation is highly correlated with the progression of myelination [62,63]. Thus, the results derived by our method (that is, the quantitative measurement of density distribution) should be affected by an age factor. As prior studies indicated, the change in brain material properties becomes clearest after the first two years of life [64,65]. Indeed, there were significant differences in the density distribution between patients at or younger than 24 months of age and patients older than 24 months; however the two groups could not be differentiated by ${\mathrm{HU}}_{17}^{24}$. Similar results were given within the severely edematous group and mildly edematous group. The results indicate that age does affect the CT density distribution; however, it has little impact on the utility of ${\mathrm{HU}}_{17}^{24}$ as an indicator of severe edema, thus allowing the proposed method to be effective. The average HU of human edematous cerebral tissue varies from 18 to 29 [66]. The numerical value of ${\mathrm{HU}}_{17}^{24}$, therefore, implies the severity of insult from cerebral edema: 16% or greater ${\mathrm{HU}}_{17}^{24}$ can be considered as a threshold for differentiating mildly edematous subjects from the severely edematous group (Figure 3). Given the definition of the two groups (the existence of severe edema), this threshold can be inferred as a potential quantitative standard for cerebral edema.

**Figure 6:**
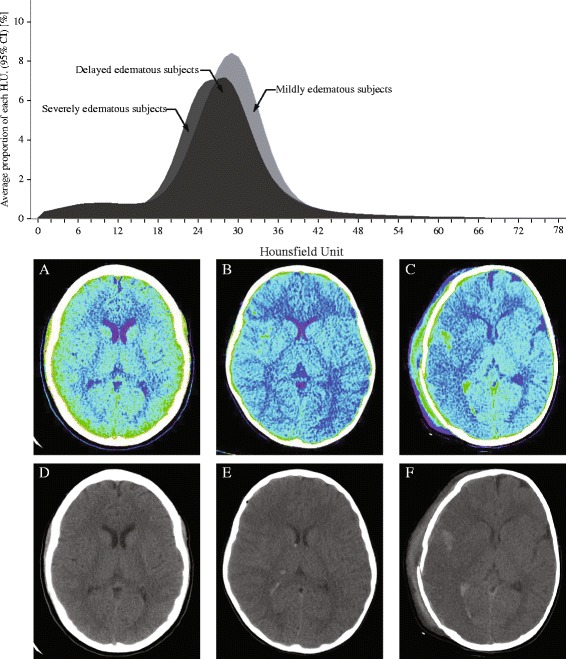
**Density histograms from initial CT images of TBI subjects.** Notice that two histograms (severe TBI subjects and subjects with delayed cerebral edema) show different distributions from mild TBI subjects. Color-mapped CT images are from **(A)** a mild TBI subject, **(B)** a subject who developed delayed cerebral edema, and **(C)** a severe TBI patient. **(D)**, **(E)**, **(F)** are their corresponding raw CT images. CT, computed tomography; TBI, traumatic brain injury.

If ${\mathrm{HU}}_{17}^{24}$ can be used to detect the presence of severe cerebral edema, the remaining question is whether it can be used to predict the development of severe cerebral edema. Delayed cerebral edema can cause delayed intracranial hypertension, which can be fatal. There were eight subjects who had no signs of severe cerebral edema in their initial CT images but later developed severe cerebral edema. If the images from the initial CT examinations of those eight subjects share a similar density distribution with the rest of the severe edema subjects, the early detection of cerebral edema would be possible. The basis for this assumption is that the initial non-enhanced brain CT images possess significant prognostic value regarding TBI [47]. Tests on subjects revealed that ${\mathrm{HU}}_{17}^{24}$ was able to differentiate the two groups successfully (Figures 4 and 6). Apparent signs of cerebral edema in a CT image become visible several hours after TBI in delayed edema subjects. However, the abnormal density distribution, expressed in HU, was already observable in their initial CT images (Figure 6). Although cytotoxic edema causes major damage in TBI, the initial edematous insult is vasogenic [67]. Evolving vasogenic edema affects the white matter. According to an earlier study, a white matter density decrease of more than 5 HU is associated with symptoms of cerebral edema [68]. However, such a subtle decrease is nearly impossible to perceive with the human eye [29]. Higher ${\mathrm{HU}}_{17}^{24}$ in the initial CT images from delayed cerebral edema subjects may reflect the early, elusive signs of vasogenic insult from TBI. This study concluded that the value of ${\mathrm{HU}}_{17}^{24}$ can predict or assess the severity of cerebral edema. Hence, ${\mathrm{HU}}_{17}^{24}$ is, hereafter, designated as the CES.

### Determinative power of cerebral edema score on mortality

Pediatric mortality is highly correlated with cerebral edema. This study hypothesized, therefore, that the CES of deceased subjects would be significantly higher than the CES of surviving subjects. Five subjects died after arrival at the hospital. The average density distribution in the initial CT images of the deceased subjects was obtained to find a correlation between mortality and CES. The CES was able to differentiate the two groups, but ${\mathrm{HU}}_{19}^{23}$ showed an even higher statistical significance. Hence, ${\mathrm{HU}}_{19}^{23}$ was considered the mCES. The difference between mCES ${\mathrm{HU}}_{19}^{23}$ and CES ${\mathrm{HU}}_{17}^{24}$ was found to be small (the proportion of pixels with HU = 18 and 24). This difference can be considered as the effect of the variance caused by a small sample size. The predictive value of ${\mathrm{HU}}_{17}^{24}$ in initial CT images for mortality should further be tested. Nevertheless, the method used in this study proved its validity in differentiating the deceased and the surviving subjects.

### Cerebral edema score as a complementary CT classification system for pediatric TBI patients

Currently, there is no CT classification system devised for pediatric patients. Instead, the Marshall classification is being employed for pediatric patients [69] as an alternative. However, this classification system does not consider cerebral edema. This limitation of the Marshall classification, combined with its high inter-observer variability [70], might result in an erroneous therapeutic approach when applied to pediatric patients. CES and mCES were developed based on CT images of pediatric TBI patients. Most importantly, these scores provide quantitative information about the level of edematous insult. These features can compensate for the previously discussed limitations of the current CT classification systems. The acquisition of CES and mCES is a simple, reproducible process that can be performed within a minute.

### Limitations and suggestions

The method devised and used for this study can be a valuable research tool in studying different aspects of TBI, including the progression of cerebral edema after initial insult, correlation between the severity of cerebral edema and clinical presentation and changes in cerebral edema in response to different therapies. Automated densitometry analysis is advisable for large-scale image analysis studies. Nevertheless, several limitations exist in the findings of this study. The patient sample size is relatively small and is limited to pediatric patients. The application of the methods used for this study for adult patients remains untested. Additionally, the method proposed in the study does not solve the problem of HU fluctuations due to the use of different CT acquisition parameters or different CT scanners. Further research is necessary to address these issues. Given these limitations, we advise using this standard to supplement existing classification systems, such as the Marshall classification, for pediatric patients.

## Conclusions

This study showed the possibility of utilizing the proportion (not number) of pixels for quantitative CT image analysis. Automated artifact elimination and densitometric measurement were introduced as tools for this analysis. Density distributions of individual patients or groups of patients can be obtained in only seconds or in a few minutes. Statistical analyses can be applied to the average density distributions between patient groups. We derived a quantitative standard (CES) for assessing the severity of cerebral edema by utilizing these methods. The proportion of pixels with HU = 17 to 24 was revealed as the key factor and, hence, was defined as the CES. A CES higher than 16% in a set of images was highly correlated with severe edema. This variable has potential as the standard for damage assessment in TBI and may even be able to serve as an early predictor of severe cerebral edema. A mCES (the proportion of pixels with HU = 19 to 23) higher than 15% can be an effective predictor for mortality. CES and mCES can be obtained by simple densitometric measurements. The acquisition of these variables can be fully automated and be performed rapidly. The application of these standards in clinical and research settings will help to better manage and understand pediatric TBI.

## Figures and Tables

**Figure 7:**
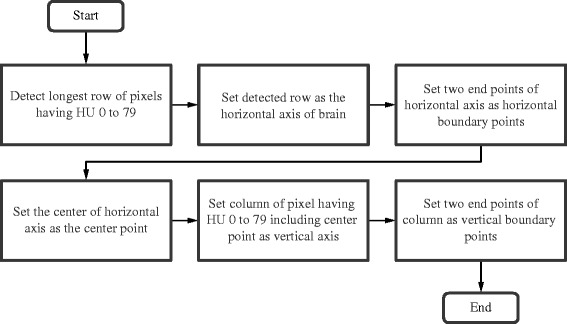
**A flowchart for assigning quadrant.**

**Figure 8:**
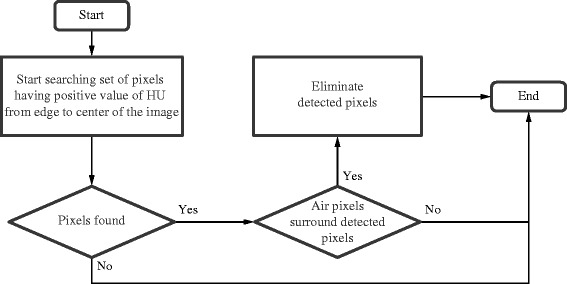
**A flowchart for the CT machine elimination.**

**Figure 9:**
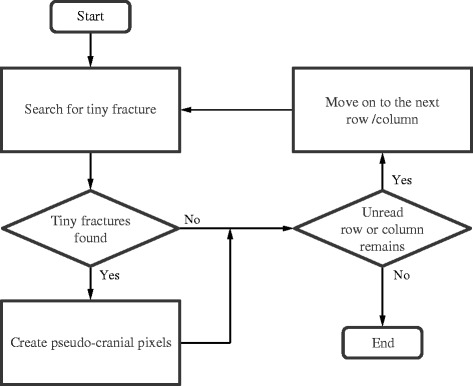
**A flowchart for sealing minor fractures.**

**Figure 10:**
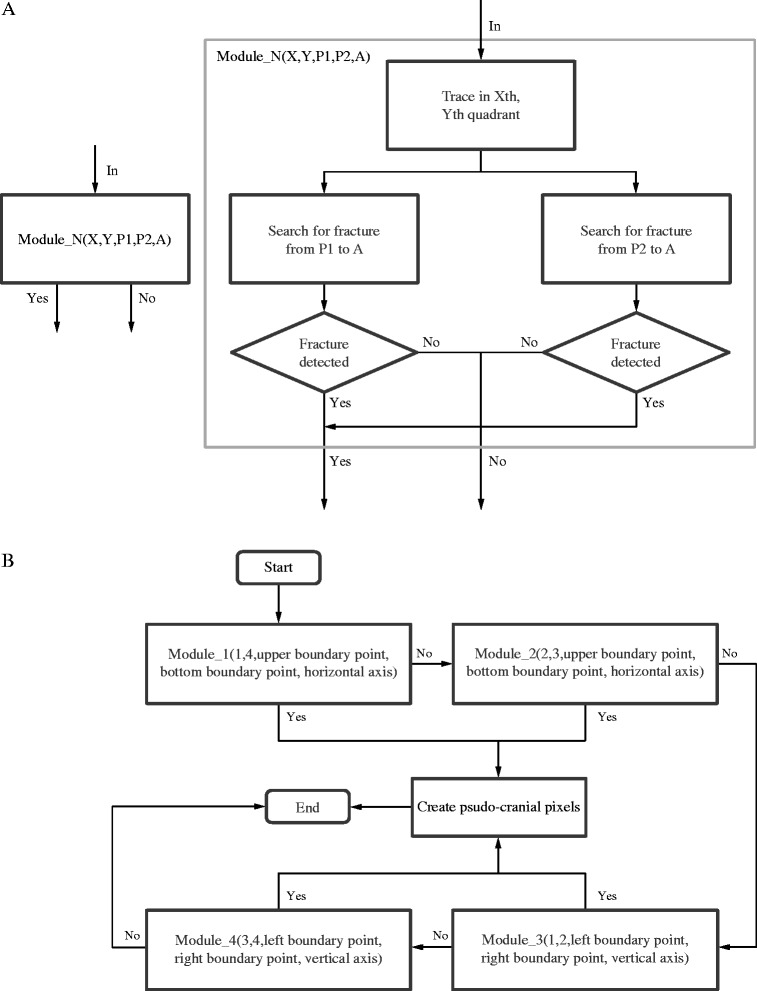
**A flowchart for sealing major fractures. (A)** A module for detecting major fractures in a single quadrant. **(B)** A flowchart for sealing major fractures using the module.

**Figure 11:**
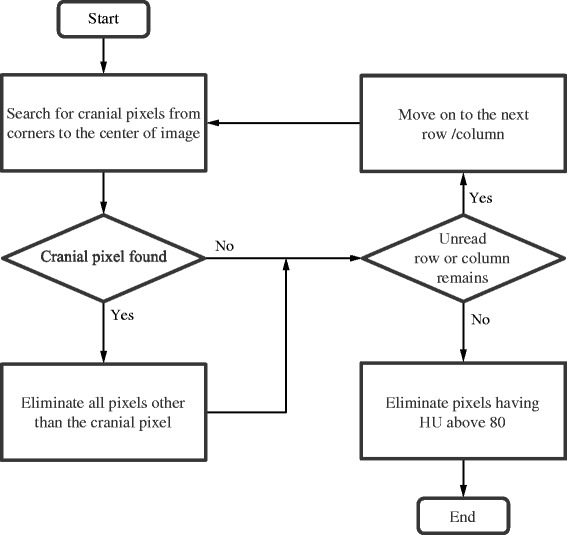
**Elimination of remaining artifacts.**

## Appendix

This section presents more details of sub-processes in the image artifact eliminating algorithm. Through a series of sub-processes, the algorithm creates an image containing only the major intracranial components (parenchyma, CSF and blood). Assigning quadrants to the intracranial area (Figure 1B), eliminating artifacts developed by the CT machine (Figure 1C), sealing the fractures across the cranium (Figure 1D) and eliminating pixels with an HU higher than 79 (Figure 1E) are the core functions of the algorithm.

Assigning quadrants to the intracranial area

All objects outside the cranium, and even the cranium itself, are considered artifacts. This definition assumes the intactness of the cranium, and hence, fractures and punctures in the cranium needed to be sealed off. This sub-process (Figure 7) exists to locate the fractures.

Elimination of pixels representing the CT machine

Pixels representing the CT machine are irrelevant to the densitometry analysis and are hence considered artifacts. To remove these pixels, the algorithm uses the pixels depicting the empty space between the CT machine and the head of the patient (Figure 8). The HU of air is approximately −1000. A set of pixels representing the CT machine is surrounded by air pixels. By this logic, the algorithm can eliminate the CT machine from a CT image.

Sealing minor fractures of the cranium

Fractures have varying sizes and locations. The algorithm separates minor and major fractures according to their sizes (Figure 9). Minor fractures are defined as less than 25 pixels in longitude and are located between the edges of the fractured cranium. The algorithm seals these fractures by creating pseudo-cranial pixels. Multiple minor fractures can exist in a single CT image, so this sub-process may be executed several times.

Sealing major fractures of the cranium

Major fractures are defined as more than 25 pixels in longitude and stretch across two quadrants. These fractures are mostly caused by craniotomy. The algorithm estimates the coordinates of the lost cranial pixels based on the coordinates of the intact cranial pixels located at the opposite sides of the horizontal or vertical axis. Pseudo-cranial pixels are created along the estimated coordinates (Figure 10).

Elimination of remaining artifacts

The sole purpose of the previous sub-processes is to create a CT image without artifacts outside the intact cranium. The final sub-process can be performed only if the cranium in the CT image is intact. The algorithm eliminates any pixels with an HU outside a predefined range of interest (in this study, 0 to 79) (Figure 11). With this last step of the automated artifact elimination algorithm completed, a CT image is standardized for later analyses.

## Abbreviations

ADC: apparent diffusion coefficient
CBF: cerebral blood flow
CES: cerebral edema score
CPP: cerebral perfusion pressure
CSF: cerebrospinal fluid
CT: computed tomography
DWI: diffusion-weighted MRI
HU: Hounsfield unit
ICP: intracranial pressure
mCES: mortality-related CES
MRI: magnetic resonance imaging
QCT: quantitative CT
ROC: receiver operator characteristics
ROI: region of interest
TBI: traumatic brain injury
